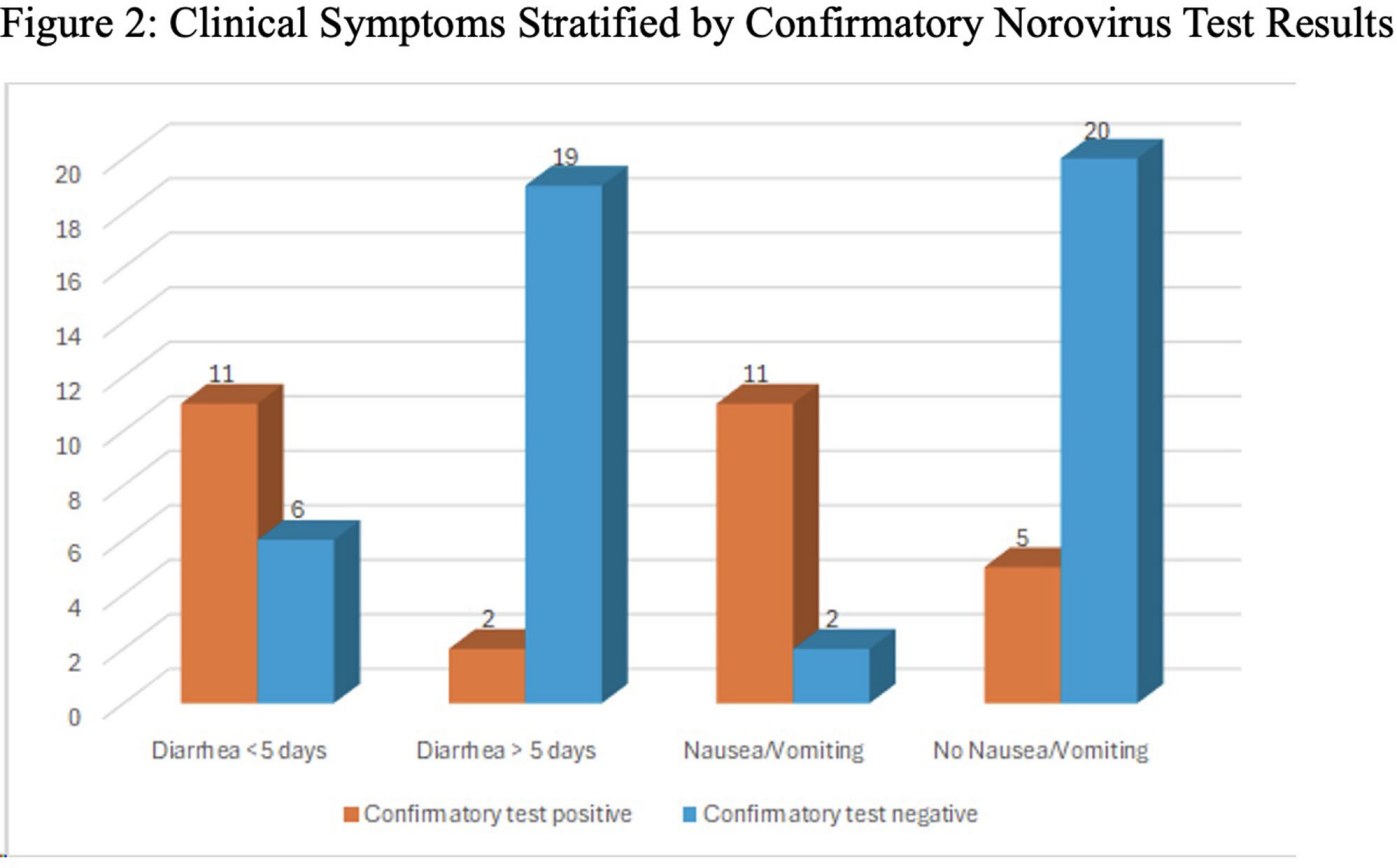# 363 Putting a Face to Hard Stop C. difficile Testing Stewardship

**DOI:** 10.1017/ash.2026.10699

**Published:** 2026-06-23

**Authors:** Ellen Earle, Christopher Pfeiffer, La’Tonzia Adams, Jennifer Holmquist, Jordan Evans, Monica K Sikka

**Affiliations:** 1 Oregon Health and Science University; 2 VA Portland Healthcare System; 3 Portland VA Medical Center

## Abstract

**Background:** Many laboratories have shifted from traditional culture-based to molecular panel diagnosis of gastrointestinal (GI) pathogens for a broad array of reasons including simplicity and rapidity. The Portland VA Health Care System (VAPORHCS) implemented the Biofire® Film array® GI pathogen panel (Biofire GI panel) 05/2024. In 04/2024, a recall alert was issued regarding increased risk of false positive norovirus results alongside a recommendation to confirm positive results by another method. The aim of this quality improvement project was to evaluate the false positive rate for norovirus of this assay. **Methods:** We used microbiology laboratory records to identify all norovirus positive results on the Biofire GI panel from 05/2024-09/2025. Chart review was performed to determine confirmatory norovirus test results, testing location (outpatient, inpatient, or emergency department), compatible clinical symptoms (acute nausea/vomiting/diarrhea), and the presence of other positive stool testing results. False positive was defined as discrepant Biofire GI panel (positive) and dedicated norovirus PCR (negative) results on the same sample. **Result:** We identified 39 initial positive tests on the Biofire GI panel from 38 unique patients. One test was excluded due to specimen processing error on the confirmatory test, leaving 38 tests for analysis. Of these, 13 (34%) were confirmed as positive by single-plex PCR corresponding to a false positive rate of 66%. False positive rate stratified by test location are shown in Figure 1. Compatible symptoms stratified by test results are shown in Figure 2. Of the true positive group, 11/13 (85%) had diarrhea < 5 days compared to 6/25 (24%) in the false positive group (p<0.001). Similarly, in the true positive group, 11/13 (85%) of patients had nausea/vomiting compared to 5/25 (20/%) in the false positive group (p<0.001). An alternative enteric pathogen was identified in 8/38 (21%) cases. **Conclusion:** We found an alarmingly high rate of false positive norovirus results on the Biofire GI panel at our center, which is higher than previously reported. True positive tests were more likely to be accompanied by those that fit with typical symptoms. Positive norovirus results from the Biofire GI panel should be interpreted with caution as is recommended by the manufacturer, particularly in absence of a compatible clinical syndrome.

**Figure:**
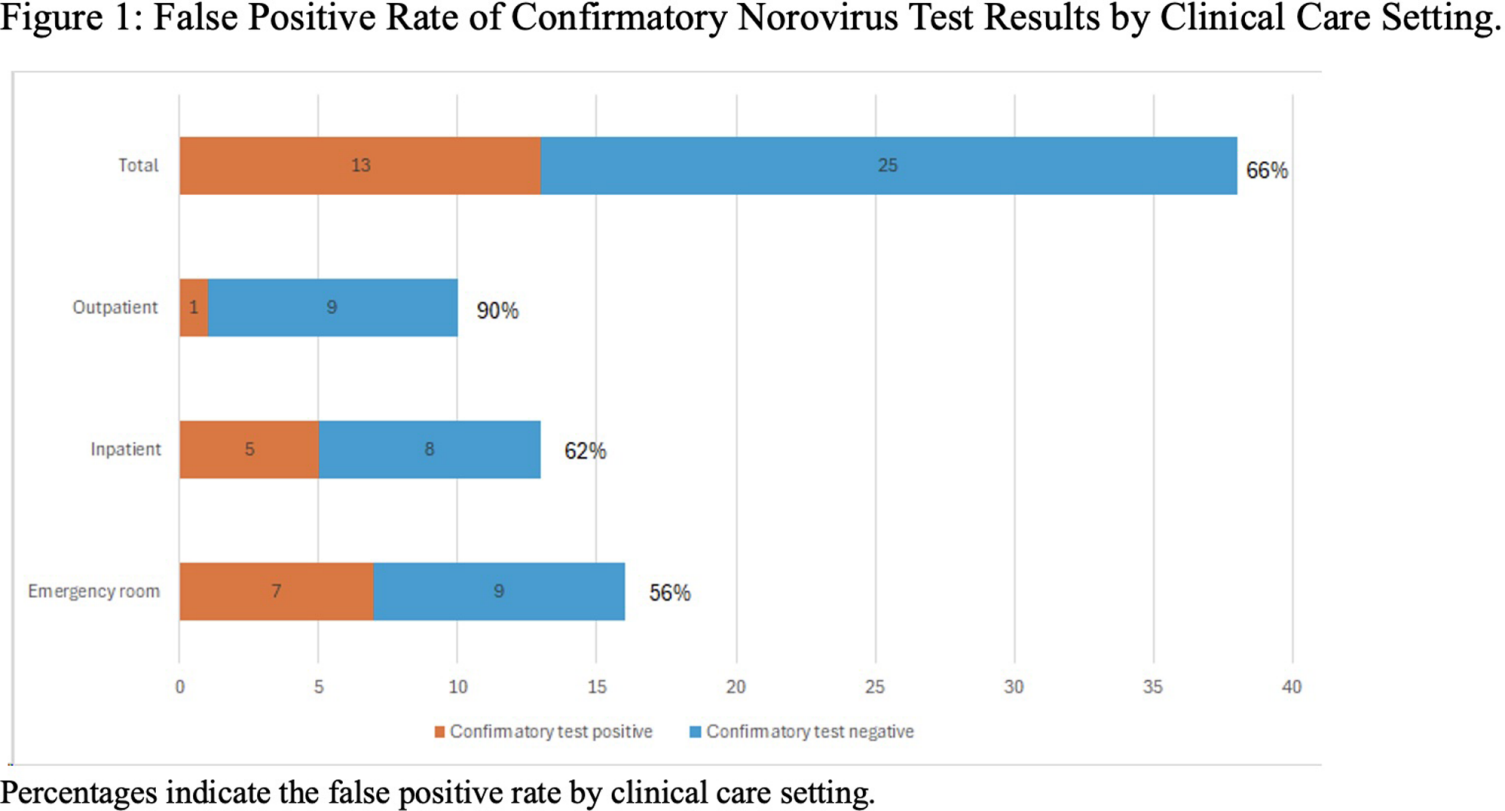

**Figure:**